# Ethnic Disparities in Clinical Decision‐Making for Rapid Tranquillisation in Adult Forensic Mental Health Inpatient Settings: A Qualitative Study

**DOI:** 10.1111/inm.70323

**Published:** 2026-07-29

**Authors:** Martin Locht Pedersen, John Baker, Trine Munk‐Olsen, Frederik Alkier Gildberg

**Affiliations:** ^1^ Forensic Mental Health Research Unit Middelfart, Department of Regional Health Research University of Southern Denmark Middelfart Denmark; ^2^ Psychiatric Department Middelfart Lillebaelt Hospital, Region of Southern Denmark Middelfart Denmark; ^3^ School of Healthcare University of Leeds Leeds UK; ^4^ Research Unit Children and Adolescent Psychiatry, Department of Clinical Research University of Southern Denmark Odense Denmark

**Keywords:** chemical restraint, clinical decision‐making, ethnicity, forced medication, forensic psychiatry, rapid tranquillisation

## Abstract

Rapid tranquillisation is a widely used restrictive practice, but evidence suggests that ethnic minorities are disproportionately exposed compared with majority populations. This retrospective qualitative multiple‐case study explored how staff clinical decision‐making regarding rapid tranquillisation, as reflected in clinical documentation, may vary by ethnicity in adult forensic mental health inpatient settings. Nineteen cases involving rapid tranquillisation use between 01 Jul 2021 and 31 Jan 2025 were purposively selected using paper‐based forensic registration forms and electronic health records. Cases were categorised dichotomously by ethnicity using country of birth (ethnic minorities vs. the majority population), and data from clinical documentation were analysed using content analysis, guided by a four‐component framework of clinical decision‐making, examining similarities and differences within and between cases. The SRQR standards were used for reporting. Overall patterns of rapid tranquillisation use were broadly similar across groups. However, differences were reported in the temporal unfolding of situations leading to rapid tranquillisation, negotiation and planning processes and the documentation of distress, severity and risk. Escalating distress and behaviour tended to be recognised later among ethnic minorities, often shortly before rapid tranquillisation, whereas escalation among the majority population was more frequently documented over longer periods. Documentation of proactive planning, voluntary medication negotiation and debriefing was limited overall but less visible for ethnic minorities. Clinical decision‐making appeared iterative and cyclical, with repeated rapid tranquillisation use occurring across both groups. Ethnicity may influence clinical decision‐making regarding rapid tranquillisation, with disparities emerging through cumulative, process‐oriented variations in practice. Further research is required to confirm these findings.

## 1 Introduction

Rapid tranquillisation (RT) is a common restrictive practice in adult mental health care internationally (Belayneh et al. 2024; Muir‐Cochrane, Grimmer, et al. 2020). It involves the forced administration of sedatives to manage acute disturbed behaviour (National Institute for Health and Care Excellence 2015, Council of Europe 2017). Although legally permitted as a last resort (Steinert and Lepping 2009), reducing RT use is a global priority due to associated risks, including injuries and serious adverse events (Muir‐Cochrane, Grimmer, et al. 2020, Muir‐Cochrane, Oster, et al. 2020, National Institute for Health and Care Excellence 2015). Notably, evidence suggests that ethnic minorities are disproportionately subjected to RT compared with majority populations (Pedersen et al. 2025a, 2025b), raising issues about equity, cultural competence and clinical decision‐making in mental health services.

RT requires staff to balance safety with respect for individual autonomy (Birkeland et al. 2024). Such decisions are often made under conditions of clinical variability and rely on nuanced clinical judgement shaped by various factors (Pedersen et al. 2024; Völlm and Nedopil 2016). In forensic mental health services, which provide specialist care for individuals with mental illness who have committed offences, this complexity is intensified. These high‐security services are characterised by legal demands and heightened risk of conflict, which may influence the likelihood and nature of RT use (Völlm and Nedopil 2016). Although many countries have expanded and re‐evaluated their forensic services (Mundt et al. 2021; Tomlin et al. 2021), evidence supporting their effectiveness remains limited (Kennedy 2022; Tully et al. 2024).

The overrepresentation of ethnic minorities in forensic populations further amplifies these concerns outlined above (Brooks et al. 2025; Smith and Mohan 2024; Völlm and Nedopil 2016). Delivering care in multicultural societies remains challenging, particularly in addressing ethnic disparities. These disparities may reflect structural and institutional biases that undermine culturally appropriate, patient‐centred practice (Buongiorno et al. 2025; Craig et al. 2025; Macpherson 1999; Pedersen et al. 2024; Williams and Etkins 2021). Evidence indicates that, compared with majority populations, ethnic minorities often experience higher rates of involuntary admission (Anderson et al. 2014; Barnett et al. 2019), longer delays and hospital stays (Ajnakina et al. 2020; Schoer et al. 2019), greater exposure to restrictive practices (Beames and Onwumere 2022; Della Rocca et al. 2024; Pedersen, Gildberg, Baker, et al. 2022), and higher risk of death associated with these practices (Aiken et al. 2011; INQUEST 2023). Staff perceptions of ethnic minorities as more dangerous (Brooks et al. 2025; Hallett et al. 2025; Jones et al. 2025) may also contribute to disproportionate RT use (Pedersen et al. 2025a, 2025b).

Explanations for these disparities in RT use often focus on individual‐level factors, such as age, gender or diagnosis (Beames and Onwumere 2022; Pedersen et al. 2025b), yet these do not fully account for observed differences (Smith et al. 2022). Staff perceptions of cultural background and language may also influence clinical decision‐making (Buongiorno et al. 2025; Della Rocca et al. 2024; Pedersen et al. 2025a, 2025b), with evidence suggesting that shared background reduces RT use (Collazos et al. 2021). Furthermore, RT remains more frequent among ethnic minorities even after adjusting for physical size as a factor influencing perceived risk of violence (Smith et al. 2022). Despite calls to strengthen the evidence base on RT and reduce restrictive practices (Muir‐Cochrane and Oster 2021; Muir‐Cochrane, Oster, et al. 2020; Pedersen et al. 2023), limited research has examined how staff make decisions about RT use in forensic services.

To address this gap, this study explored how staff clinical decision‐making regarding RT, as reflected in clinical documentation, may vary by ethnicity in adult forensic mental health inpatient settings. Understanding these dynamics could enhance cultural competence, support safer and more equitable care, and reduce RT reliance.

## 2 Methods

This retrospective qualitative study employed a multiple‐case framework inspired by Baxter and Jack (2008) and was grounded in a pragmatist methodology following Blumer (1986). This approach is well‐suited to examining clinical decision‐making in forensic services, which may be complex and context‐dependent, allowing for a deeper exploration of RT use among individuals from diverse ethnic backgrounds. The research team consisted of individuals with nursing backgrounds with varying academic qualifications, half of whom had forensic clinical experience. All members were external to the research context, with no prior clinical contact with individuals. The study was reported following the Standards for Reporting Qualitative Research (SRQR; Supporting Information) (O'brien et al. 2014) and addressed the following research question:

- What characterises staff clinical decision‐making regarding RT use in individuals from ethnic minority backgrounds in adult forensic mental health inpatient settings compared with those from the majority population?

### 2.1 Setting and Sample

The study was conducted at the largest hospital site covering adult (≥ 18 years old) forensic mental health inpatient settings in the Region of Southern Denmark. Cases involving individuals subjected to RT between 01 Jul 2021 and 31 Jan 2025 were purposively selected (Baxter and Jack 2008) based on the following inclusion criteria:

- Availability of a paper‐based registration form for the Danish Forensic Mental Health Database (sundk.dk).
- Documented RT use in electronic health records.

Cases that did not meet these criteria were excluded. For individuals with repeated RT use, only the first available case within the study period was included, ensuring that each case could be analysed independently in line with the multiple‐case framework (Baxter and Jack 2008).

In Denmark, according to the Mental Health Act (Ministry of the Interior and Health 2024), RT may be administered (e.g., orally or intramuscularly) following a medical assessment if deemed essential for improving the condition of a highly agitated individual. The start date of 01 Jul 2021 was selected to align with a significant shift in Danish regulations covering electronic health record documentation (Ministry of the Interior and Health 2021), thereby ensuring a consistent data foundation throughout the study period. Consistent with prior research on restrictive practices in forensic services (Gildberg et al. 2015), each case encompassed the period shortly before, during and shortly after RT use (±3 days), and was intended to enable the exploration of similarities and differences within and between cases (Baxter and Jack 2008). Sample size was determined considering information richness within the constraints of the available dataset (Morse et al. 2002).

**FIGURE 1:**
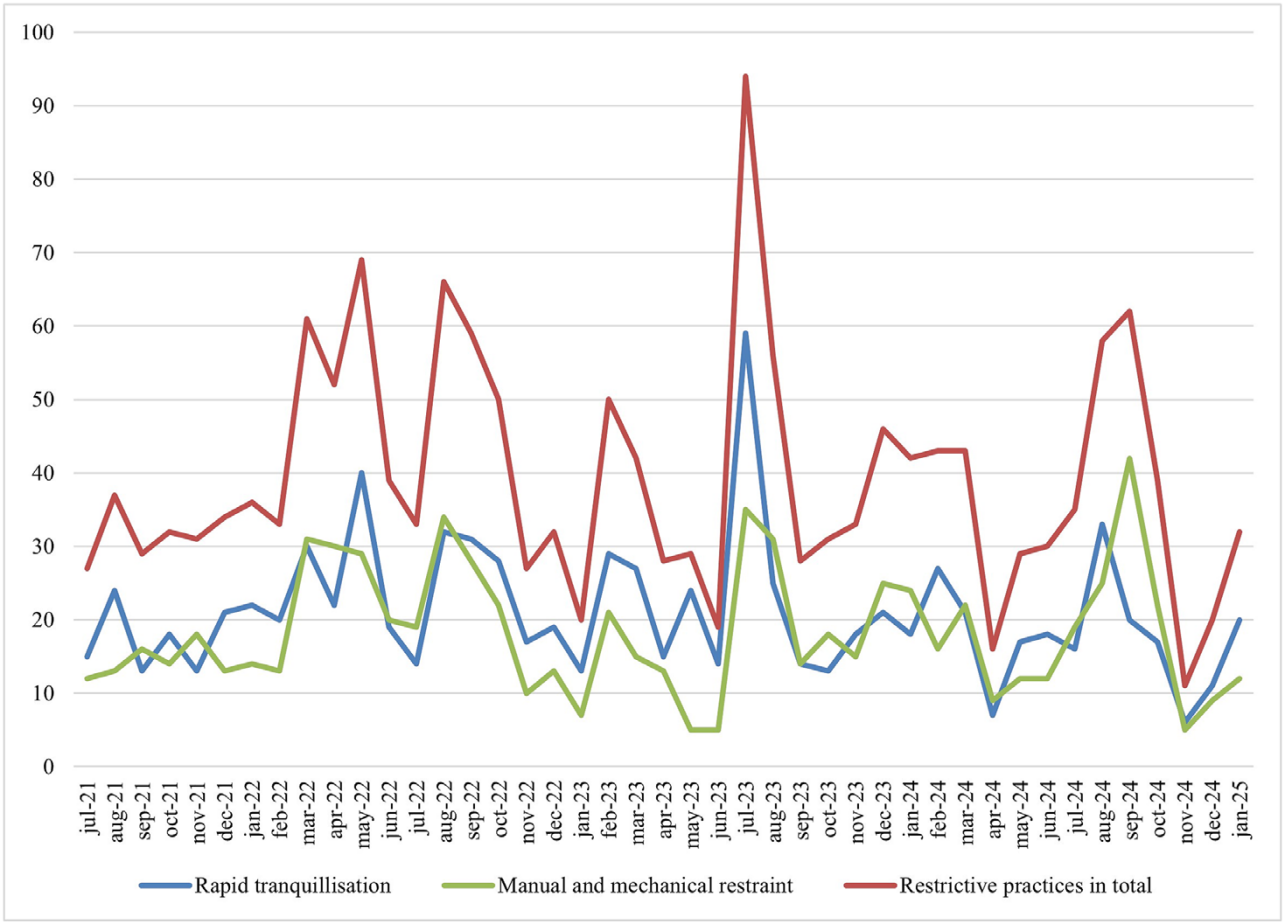
Cases involving rapid tranquillisation, manual and mechanical restraint and total cases of these restrictive practices, at the largest forensic hospital site within the Region of Southern Denmark between 01 Jul 2021 and 31 Jan 2025.

During the study period visualised in Figure 1, 901 RT cases occurred, representing 54.2% of all restrictive practice use (RT, manual and mechanical restraint; *N* = 1683). These cases involved 100 individuals, of whom 19 (19%) met the inclusion criteria outlined above and were included in the study.

### 2.2 Data Collection

We incorporated data from registration forms for the Danish Forensic Mental Health Database and electronic health records. Registration forms are completed only for individuals subject to a forensic mental health measure; further information on these measures in Denmark is available elsewhere (e.g., Pedersen, Gildberg, and Birkeland 2022). The two data sources were linked using the Danish civil registration number (CPR) and recorded in an Excel spreadsheet via SharePoint.

Cases were categorised dichotomously into two ethnic groups according to country of birth, the available operationalisation of ethnicity in the data extracted from the registration forms: ethnic minorities (individuals born abroad, i.e., in Western countries, the Middle East, Africa (other), the Rest of the World, Greenland or with unknown country of birth; *n* = 7) and the majority population (individuals born in Denmark; *n* = 12).

For each included case, all available clinical documentation (±3 days surrounding RT use) was extracted from the electronic health records, including structured fields (e.g., medication lists, restrictive practice records, risk assessments) and free‐text notes (e.g., staff observations, behavioural descriptions, interactions).

To describe the cases, additional data were extracted from registration forms, including characteristics of included individuals (e.g., age at sentencing, gender, forensic measure, legal status), while total RT use during the study period was extracted from the electronic health records.

Case overviews are shown in Tables 1 and 2.

**TABLE 1:** Characteristics of included individuals.

	In total (*N* = 19)	Ethnic minorities (*n* = 7)	Ethnic majorities (*n* = 12)
Age at sentencing, median (IQR)	30 (24–38)	27 (23–32)	34 (27–39)
Gender, *n* (%)			
Female	5 (26)		
Male	14 (74)		
History of substance use, *n* (%)			
Alcohol	6 (32)	3 (43)	3 (25)
Cannabis	11 (58)	4 (57)	7 (58)
Other	8 (42)		
No or unknown	6 (32)		
Forensic measure for mandatory treatment, *n* (%)	19 (100)	7 (100)	12 (100)
Types of offence (s), *n* (%)			
Assaults against public officials	9 (47)		
Other violent offences and threats	10 (53)	5 (71)	5 (42)
Other criminal offences	8 (42)	4 (57)	4 (33)
Other	5 (26)		
History of clonvictions, *n* (%)			
Ordinary sentence	9 (47)		
Forensic measure	4 (21)		
No	6 (32)		
Criminal code, *n* (%)			
Para16	16 (84)	7 (100)	9 (75)
Para 69	3 (16)	0 (0)	3 (25)
Time limitation of sentencing, *n* (%)			
Yes	8 (42)		
No	11 (58)		
Sentence without expulsion, *n* (%)	19 (100)	7 (100)	12 (100)
Living situation, *n* (%)			
Own residence	13 (68)		
Residential care or homeless	6 (32)		
Civil status single or unknown, *n* (%)	19 (100)	7 (100)	12 (100)
Income source, *n* (%)			
Pension or disability pension	13 (68)	4 (57)	9 (75)
Other transfer income or unknown	6 (32)	3 (43)	3 (25)
Psychopharmacological treatment, *n* (%)			
Antipsychotics Benzodiazepines	15 (79) 3 (16)	0 (0)	3 (25)
Other	5 (26)	0 (0)	5 (42)
No	4 (21)		

*Note:* Ethnic minorities: individuals born abroad; Ethnic majorities: individuals born in Denmark. Para 16: legally not responsible due to severe mental disorder; Para 69: less severe impairments, courts may impose forensic measures instead of punishment. Variables were obtained from registration forms for the Danish Forensic Mental Health Database, completed around the time of sentencing. For individuals with more than one registration form available, the one completed closest prior to the selected rapid tranquillisation use was employed. Grouping of variables and blank fields was done to protect the anonymity of included individuals. Percentages do not always total 100% as individuals may appear more than once. Values were rounded to nearest whole number.

Abbreviations: IQR; interquartile range.

**TABLE 2:** Characteristics of rapid tranquillisation (RT) use.

	In total (*N* = 19)	Ethnic minorities (*n* = 7)	Ethnic majorities (*n* = 12)
Age at RT use, median (IQR)	34 (27–41)	34 (26–38)	36 (38–43)
RT medication, *n* (%)			
Antipsychotics or benzodiazepines	9 (47)	3 (43)	5 (50)
Combination	10 (53)	4 (57)	5 (50)
Administration route, *n* (%)			
Oral	9 (47)	3 (43)	6 (50)
Intramuscular	11 (58)	4 (57)	7 (58)
Concurrent manual or mechanical restraint, *n* (%)			
Yes	10 (53)	3 (43)	7 (58)
No or unknown	9 (47)	4 (57)	5 (42)
Reference to previous RT effect, *n* (%)			
Yes	4 (21)		
No	15 (79)		
RT use without reference to advance statement, *n* (%)	19 (100)	7 (100)	12 (100)
Number of RT use during the study period, median (IQR)	3 (1–7)	2 (2–7)	3 (1–7)

*Note:* Ethnic minorities: individuals born abroad; Ethnic majorities: individuals born in Denmark. Variables were obtained from electronic health records around the time of RT use. Grouping of variables and blank fields was done to protect the anonymity of included individuals. Percentages do not always total 100% as individuals may appear more than once. Values were rounded to nearest whole number.

Abbreviations: IQR; interquartile range.

### 2.3 Analysis

Cases were analysed using content analysis (Gildberg and Wilson 2023a, 2023b; Krippendorff 2004). To explore staff clinical decision‐making regarding RT use, data from clinical documentation were categorised according to the four‐component framework of clinical decision‐making in RT use in adult mental health inpatient settings previously identified and synthesised in a review by Pedersen et al. (2023): becoming aware of situational changes and considering alternatives, negotiating voluntary medication, administering RT and being on the other side.

The framework was used as an analytic structure to organise and compare data across cases, capturing the period shortly before, during and shortly after RT use (±3 days). Data were initially coded through systematic reading of all available clinical documentation, identifying segments of text reflecting clinical decision‐making, which were subsequently categorised within the four components of the framework. Within each component, similarities and differences were examined within and between cases (Baxter and Jack 2008), with particular attention to ethnic minorities and the majority population.

Analysis was conducted by the first author. Data quality and interpretative rigour were ensured through ongoing peer discussions within the research team, fostering trustworthiness and credibility throughout the process (Morse et al. 2002).

## 3 Results

### 3.1 Characteristics of Included Cases

As shown in Tables 1 and 2, 19 cases were available for inclusion in the study. Most cases involved males, with a high prevalence of substance use history, particularly cannabis. In all cases, individuals were subject to a forensic mandatory care and treatment order and had primarily committed violent offences, with approximately two‐thirds having prior convictions. RT was administered via both monotherapy and combination therapy, using oral or intramuscular routes, and in several cases, individuals had a history of repeated RT. Overall patterns were broadly similar across ethnic groups, with minor differences in, for instance, age at sentencing, substance use and RT frequency.

### 3.2 Results of Content Analysis

**FIGURE 2:**
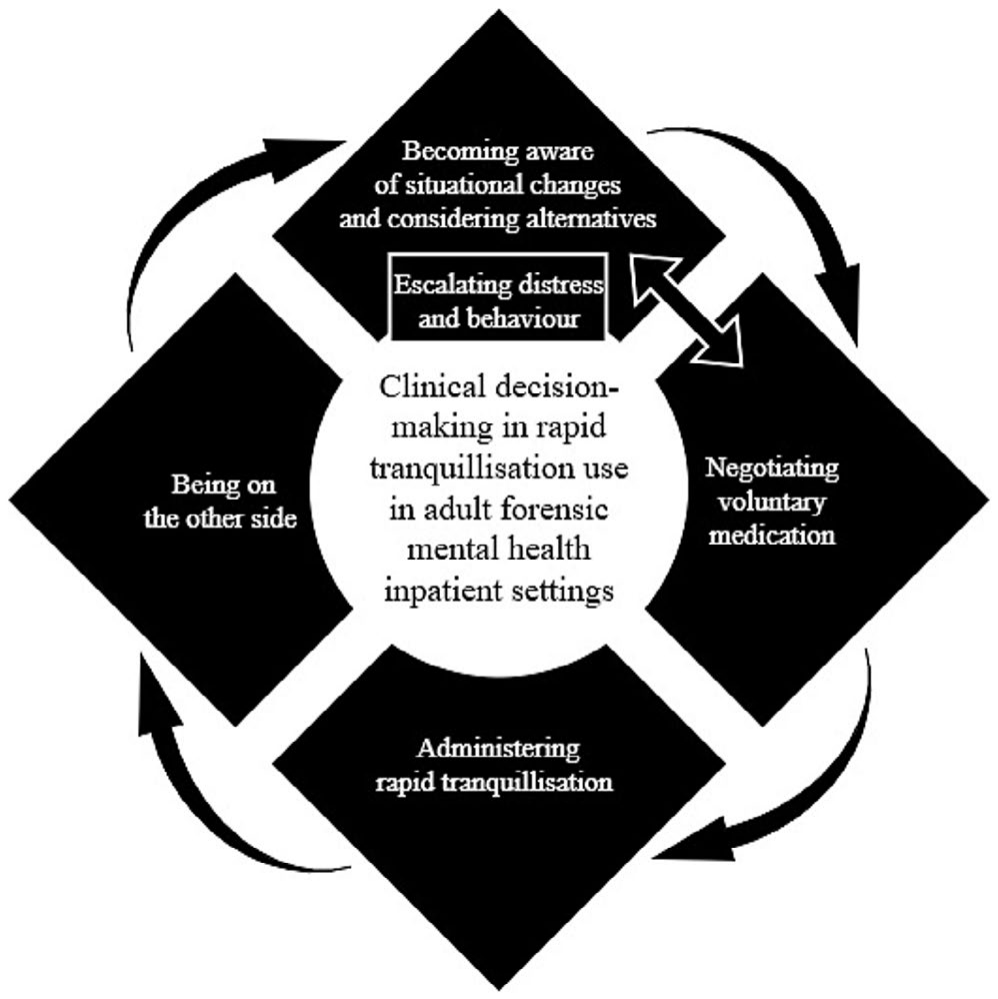
Visualisation illustrating components of staff clinical decision‐making in rapid tranquillisation use in adult forensic mental health inpatient settings.

Analysis revealed patterns in staff clinical decision‐making around RT, structured according to the four components of Pedersen et al. (2023). While these components are presented sequentially (Pedersen et al. 2023), our analysis highlighted a more circular and iterative process in practice. Staff frequently moved back and forth between becoming aware of situational changes, considering alternatives and negotiating voluntary medication, rather than following a linear path toward RT. Repeated RT use within the days shortly after the first episode in several cases indicated a cyclical dynamic over time. Additionally, the first component – ‘becoming aware of situational changes and considering alternatives’ – revealed distinct temporal escalation patterns, leading us to add a sub‐component capturing escalating distress and behaviour. A visual representation of this updated framework is provided in Figure 2.

#### 3.2.1 Becoming Aware of Situational Changes and Considering Alternatives

RT often reflected situational changes occurring well before administration, making a clear starting point difficult to identify. High levels of agitation and aggression, such as property damage prompting RT, were documented as individual responses to disagreement with the grounds for admission. Ethnic minorities were generally admitted only a few days before RT, often following non‐adherence to treatment or agreements with authorities, behavioural changes, and documented criminal recidivism, including multiple recent police contacts. Police‐assisted admissions were noted in several cases. The majority population exhibited similar patterns but were usually admitted for longer periods prior to the first administration of RT.

Clinical presentations at admission varied. Some individuals appeared calm, while others had complex needs or substance use issues. Several expressed dissatisfaction, requested immediate discharge or sought to file complaints. All ethnic minorities were assessed as medically impaired and mostly unwilling to resume treatment; nevertheless, medication was prescribed, often based on prior prescriptions. Among the majority population, some individuals had adhered to treatment plans until admission. Overall, acceptance of the situation was more common among the majority population, whereas dissatisfaction was more evident among ethnic minorities.

##### 3.2.1.1 Escalating Distress and Behaviour

Despite heterogeneous initial presentations, documentation revealed a recurring pattern of escalating distress leading up to RT, driven by individual behaviour. Escalation could occur suddenly or evolve over time. Among the majority population, escalation was often observable several days in advance, whereas for ethnic minorities, it typically emerged only hours before RT. Common behaviours included sleep deprivation, irritability, fearfulness, and uncooperativeness. Escalation also sometimes involved psychotic statements, provocations, and threats toward staff or others, such as arson. Self‐harm was documented only among the majority population. Communication difficulties were noted in a few cases involving ethnic minorities.

These situational changes led to staff initiating preventive, non‐pharmacological measures, such as quiet, low‐stimulation environments, verbal de‐escalation, risk assessment, and spatial distancing. Escalation triggers often included refusals of requests following daily activities, including medical reviews. Rapid response teams were occasionally called to support treatment plans, and alarms were activated to increase staffing. No systematic differences in staff measures were documented between ethnic groups; however, staff frustration was more often documented toward the majority population, occasionally reducing de‐escalation effectiveness. Proactive planning, including suicide risk and substance withdrawal screening and advance statements, was also documented more frequently for the majority population.

#### 3.2.2 Negotiating Voluntary Medication

Negotiation of voluntary medication was an ongoing process addressing both acute behavioural management and general treatment. Non‐adherence was a common reason for escalation, described commonly among ethnic minorities as a lack of service engagement and involvement, and among the majority population as recent refusal or lack of perceived effect. Medication was offered either orally or intramuscularly, with staff encouraging calm, collaborative acceptance. Some individuals requested medication early in escalation, often for specific drugs, though consent was occasionally withdrawn later. The majority population was generally more accepting of medication during the negotiation period, often viewing it as a step toward discharge; however, documentation of negotiations was generally limited, particularly for ethnic minorities.

At times, staff assessed that severe agitation necessitated RT, often prompting alarm activation or contact with medical staff, if not already present, to (re‐) establish a treatment plan. While no systematic differences were recorded between ethnic groups, proactive planning, such as acute medication strategies, was more frequently documented for the majority population. RT decisions were reported as collaborative and considered individual conditions, as well as prior medication experiences in few cases, which occasionally limited treatment options, particularly for ethnic minorities. Although advanced statements were more often obtained and risk assessment strategies frequently recorded for the majority population, their influence on management of situations was limitedly documented for either group.

#### 3.2.3 Administering RT

RT was most often administered in the afternoon or evening for ethnic minorities, whereas timing for the majority population was less consistent, with morning and night administrations also common. Staff offered some individuals a choice of administration route, aware that RT would be recorded as involuntary regardless of route, preserving the right to complain. In a few cases, particularly among the majority population, RT was administered via injection due to non‐cooperation with oral administration. RT was frequently accompanied by restraint following staff assessments of agitation and risk, with some individuals from the majority population having already been restrained for hours at administration. However, RT was also sometimes administered calmly, even when individuals were verbally abusive or intended to file complaints.

Police often assisted with dialogue and restraint in cases involving a history of violence. Their presence sometimes facilitated cooperation, with no systematic differences between ethnic groups. Behaviour during RT varied widely, ranging from mood shifts, fighting, spitting, or shouting (often directed at police when present) to disengagement, avoiding eye contact, and passively accepting the administration involuntarily. Staff provided verbal and written information about RT and individual rights. In addition to police presence, staffing during RT was often increased through personnel responding to alarms. Documentation more frequently characterised RT among ethnic minorities as severe, although some of the most violent case descriptions involved the majority population.

#### 3.2.4 Being on the Other Side

The post‐RT period typically began immediately after administration. Only a few cases involving ethnic minorities were formally debriefed, with minimal indication of intent to file complaints. Similar patterns were reported for the majority population, although some individuals expressed guilt, shame, or frustration, often without documented staff response. Contact with patient advisors was rare recorded but slightly more common among the majority population, typically involving those who had also been restrained and mainly relating to restraint. Some individuals were largely unresponsive to RT, particularly if still restrained, with attention often focused on restraint duration. Documented behaviours across ethnic groups included mood shift, ambivalence about medication, repeated activity requests, and nickname use. Staff primarily prioritised immediate safety while balancing individual needs and procedural requirements, focusing on maintaining calm, providing care, offering voluntary medication, and encouraging treatment continuation until release.

In some cases, additional restraint was required shortly after RT due to insufficient medication effect or heightened anger, including threats or dissatisfaction toward staff or others, particularly among some individuals from the majority population. Staff employed de‐escalation strategies like those used earlier, including non‐pharmacological measures, risk assessment, additional medication, increased staffing or rotating primary contact staff, while continually assessing the need for further situation management. Repeated RT following initial administration occurred across ethnic groups in several cases. Although this was often documented among ethnic minorities, some of the highest recurrence rates were reported among the majority population, where repeated RT was sometimes documented to minimise restraint duration. Other individuals coped with the post‐RT period by withdrawing, isolating, or attempting to calm down or sleep.

## 4 Discussion

This study explored how staff clinical decision‐making regarding RT use was documented and appeared to unfold in adult forensic mental health inpatient settings, with particular attention to potential ethnic disparities. Overall patterns of RT use, including medication type, administration route and co‐occurring restraint, were broadly similar across ethnic groups. However, equality in such recorded outcomes did not equate to equality in process. Important differences were identified in the temporal unfolding of situations, negotiation and planning processes and the documentation of distress, severity and risk. Ethnic disparities thus appeared to emerge through cumulative, process‐oriented variations in institutional trajectories, highlighting the importance of examining RT within the broader context of forensic services.

Applying and adapting the four‐component framework proposed by Pedersen et al. (2023), our analysis demonstrates that clinical decision‐making surrounding RT is not linear but iterative and cyclical. Staff frequently moved back and forth between becoming aware of situational changes, considering alternatives and negotiating voluntary medication. The frequent occurrence of repeated RT illustrates how cycles of restrictive practices may be sustained over time rather than resolving acute disturbed behaviours definitively. These findings align with previous research describing similar self‐reinforcing patterns in which multiple restrictive practices are applied repeatedly or concurrently (Baker et al. 2021; Müller et al. 2023; Pedersen, Gildberg, Baker, et al. 2022). In our study, this pattern was also reported in relation to restraint. Importantly, incorporating a sub‐component capturing escalating distress and behaviour refines the framework by acknowledging that clinical decision‐making regarding RT use may be shaped by gradual or sudden escalation, providing a more accurate representation of the complexity of RT use in forensic services, as suggested by others (Völlm and Nedopil 2016).

Escalation leading to RT was often recognised later among ethnic minorities, typically hours rather than days before administration, compared with the majority population. This compressed recognition window may reduce staff opportunities for, for instance, measures such as de‐escalation (Patel et al. 2018), thereby positioning ethnic minorities on a faster, more acute process toward RT. Documentation of voluntary medication negotiation and proactive planning, including risk assessment and advance statements, also appeared less visible for ethnic minorities. These patterns suggest that ethnic disparities may not stem from single decision points but instead accumulate across multiple, interconnected institutional impact points within forensic services, shaping the timing, visibility and options available to different ethnic groups in clinical decision‐making regarding RT (Pedersen et al. 2023).

Several interacting explanations may contribute to these patterns. Thus, potential explanations include cultural differences in service engagement, involvement and communication, as evident in some cases. However, ethnic disparities may not be reduced to such factors alone. Additionally, as demonstrated by others, staff‐related factors, such as limited cultural understanding and assumptions regarding adherence or perceived risk, may also contribute to ethnic disparities (Brooks et al. 2025; Collazos et al. 2021; Smith and Mohan 2024). The presence of institutional racism in mental health services is likely involved as well (Craig et al. 2025; Macpherson 1999; Williams and Etkins 2021). Consistent with prior research showing lower‐quality adult mental health care for ethnic minorities compared with majority populations worldwide (Ajnakina et al. 2020; Brooks et al. 2025; Pedersen, Gildberg, Baker, et al. 2022; Pedersen et al. 2025b), our findings provide qualitative insight into how such disparities may become embedded in routine processes.

RT use was only minimally debriefed across ethnic groups, despite evidence that debriefing may mitigate emotional impact and fear following restrictive practices (Mangaoil et al. 2020; Mckenna et al. 2024; Patel et al. 2018; Riahi et al. 2020). This is concerning given the legal requirement for staff to offer debriefing in the research context (Mckenna et al. 2024, Ministry of the Interior and Health 2024), as well as recommendations in leading international guidelines (National Institute for Health and Care Excellence 2015, Council of Europe 2017). Limited documentation may partly reflect the short case window (±3 days) or ongoing restrictive practice use post‐RT, during which individuals may not have been considered ready for debriefing. However, expressions of guilt, shame, or frustration were documented only among some individuals from the majority population, with staff responses rarely recorded, while similar expressions were absent in documentation for ethnic minorities.

This absence may not indicate a lack of emotional response among individuals but rather differences in recognition, documentation practices or recording of behavioural presentation, potentially reinforcing disparities in post‐RT support and limiting opportunities for reflection, relational repair and informing future clinical‐decision‐making surrounding RT (Patel et al. 2018; Pedersen et al. 2023). Contact with patient advisors further suggested that RT was seldom addressed post‐use, particularly when restraint had been applied. These findings indicate that debriefing should be conceptualised beyond legislative compliance (Mckenna et al. 2024) and more consistently embedded in policy, guidance and staff training, as recommended by Mangaoil et al. (2020). Importantly, our findings also highlight the need to explore why post‐RT expressions and documentation differ by ethnicity and how these disparities can be addressed in practice. The identified ethnic disparities may represent processes through which inequalities persist, not only in practice exposure but also in subsequent support.

### 4.1 Implications for Future Research

Future research should examine larger, multi‐site samples to confirm these findings and explore intersectional factors, including gender, diagnosis, substance use and social determinants, that may influence RT use between ethnic groups (Flanagin et al. 2021, 2025b; Smith and Mohan 2024). Research evaluating interventions aimed at reducing RT reliance and their impact on ethnic disparities is also needed (Baker et al. 2021). Additionally, examining post‐RT outcomes, such as patient experience, trauma, treatment adherence and physical health (Paton et al. 2019, National Institute for Health and Care Excellence 2015, Council of Europe 2017), would provide a more comprehensive understanding of the implications of restrictive practices like RT.

### 4.2 Limitations

This study has several limitations. Although the multiple‐case framework enabled analysis within and between cases (Baxter and Jack 2008), the number of cases included may limit the breadth of perspectives captured. Therefore, due to the constraints of the available dataset, nuances in clinical decision‐making related to ethnicity in adult forensic mental health inpatient settings may remain unexplored. Notably, ethnicity was categorised solely by country of birth (ethnic minorities vs. the majority population), which may have obscured important within‐group differences. For individuals with repeated RT use, only the first available case was included in line with the multiple‐case framework (Baxter and Jack 2008); however, this may have limited insights into escalation patterns or further potential ethnic disparities in repeated use. The research team, although including members with forensic clinical experience, had no prior clinical contact with the individuals. This outsider perspective reduced potential bias but may have limited access to implicit contextual knowledge (Buongiorno et al. 2025). The retrospective use of registration forms and electronic health records restricted analysis to formally documented reasoning, omitting unrecorded factors influencing RT decisions. Despite a rigorous analytic approach and team‐based discussions to enhance trustworthiness and credibility, interpretation is inevitably shaped by the perspectives and decisions made during the analysis. Finally, findings reflect a Danish legal and institutional context and may not generalise to other countries or non‐forensic settings.

## 5 Conclusions

In conclusion, this study revealed that ethnicity may influence staff clinical decision‐making regarding RT in adult forensic mental health inpatient settings, with disparities emerging through cumulative, process‐oriented variations in practice. Incorporating escalating distress and behaviour and highlighting iterative, cyclical patterns in clinical decision‐making refines understanding of RT use in forensic services. Further research is needed to examine and nuance how ethnicity intersects with other factors influencing RT use and support equitable, rights‐based care that minimises RT reliance.

### 5.1 Relevance for Clinical Practice

Efforts to address ethnic disparities in RT should focus not only on reducing overall use but also on limiting repeated use and improving processes surrounding RT. Early‐stage assessment, communication and de‐escalation appear crucial. Forensic services should prioritise recognising early warning signs of escalating distress and behaviour in ethnic minorities (Fluttert et al. 2008), using structured risk assessment, proactive planning and thorough documentation to ensure equitable timing of measures, recognising that staff familiarity may be shorter than with the majority population. Enhancing documentation of negotiation, refusal and reasoning across ethnic groups may strengthen transparency, support individual autonomy (Birkeland et al. 2024) and reduce RT reliance. Consistent with prior research on RT use (Pedersen et al. 2025a, 2025b)¸ staff training should focus on cultural competence and, importantly, cultural safety (Mcgough et al. 2022) to mitigate cumulative, process‐oriented disparities in institutional trajectories.

## Author Contributions

M.L.P.: writing – review and editing, writing – original draft, Visualisation, Software, Resources, Project administration, Methodology, Investigation, Funding acquisition, Formal analysis, Data curation, Conceptualization. J.B.: writing – review and editing, Validation, Supervision, Methodology, Conceptualization. T.M.‐O.: writing – review and editing, Validation, Supervision, Methodology, Conceptualization. F.A.G.: writing – review and editing, Validation, Supervision, Methodology, Conceptualization.

## Funding

The work of M.L.P. for this project was supported by the Novo Nordisk Foundation (NNF22OC0080419). The study's funder had no role in the study design, data collection, data analysis, data interpretation, writing of the report or the decision to submit the paper for publication.

## Ethics Statement

This study was registered with the Danish Data Protection Agency via the Mental Health Services in the Region of Southern Denmark (no. 25/1170) and approved by the Mental Health Services and the Region Council in Southern Denmark (no. 25/13430). Data were processed and stored by the European Union General Data Protection Regulation (GDPR). In Denmark, ethics committee approval is not required for this type of study. To protect individual anonymity, no detailed identifying information is provided, including direct quotes, like others (Gildberg et al. 2015), although this might have further substantiated analytic findings (O'brien et al. 2014). Information about the ongoing study was made available within the research context via a central electronic board.

## Conflicts of Interest

M.L.P. has received grants from FOSTREN; was awarded the 2025 Kirsten Stallknecht Prize by the Danish Nurses Organisation; and won the Region of Southern Denmark and Faculty of Health Sciences, University of Southern Denmark 2025 PhD Cup. T.M.‐O. has received speaker honoraria from Lundbeck A/S. F.A.G. is an Editorial Board Member of the International Journal of Mental Health Nursing. The remaining authors declare no conflicts of interest.

## Supporting information


**Data S1:** SRQR Checklist.

## Data Availability

The data supporting the findings of this study are not publicly available but may be made available from the authors upon reasonable request.

## References

[inm70323-bib-0001] Aiken, F. , J. Duxburry , C. Dale , and I. Harbison . 2011. Review of the Medical Theories and Research Relating to Restraint Related Deaths. University of Central Lancashire.

[inm70323-bib-0002] Ajnakina, O. , B. Stubbs , E. Francis , et al. 2020. “Hospitalisation and Length of Hospital Stay Following First‐Episode Psychosis: Systematic Review and Meta‐Analysis of Longitudinal Studies.” Psychological Medicine 50: 991–1001. 31057129 10.1017/S0033291719000904

[inm70323-bib-0003] Anderson, K. K. , N. Flora , S. Archie , C. Morgan , and K. Mckenzie . 2014. “A Meta‐Analysis of Ethnic Differences in Pathways to Care at the First Episode of Psychosis.” Acta Psychiatrica Scandinavica 130: 257–268. 24580102 10.1111/acps.12254 PMC4336563

[inm70323-bib-0004] Baker, J. , K. Berzins , K. Canvin , et al. 2021. “Health Services and Delivery Research.” In Non‐Pharmacological Interventions to Reduce Restrictive Practices in Adult Mental Health Inpatient Settings: The COMPARE Systematic Mapping Review. NIHR Journals Library. 33651527

[inm70323-bib-0005] Barnett, P. , E. Mackay , H. Matthews , et al. 2019. “Ethnic Variations in Compulsory Detention Under the Mental Health Act: A Systematic Review and Meta‐Analysis of International Data.” Lancet Psychiatry 6: 305–317. 30846354 10.1016/S2215-0366(19)30027-6 PMC6494977

[inm70323-bib-0006] Baxter, P. , and S. Jack . 2008. “Qualitative Case Study Methodology: Study Design and Implementation for Novice Researchers.” Qualitative Report 13: 544–559.

[inm70323-bib-0007] Beames, L. , and J. Onwumere . 2022. “Risk Factors Associated With Use of Coercive Practices in Adult Mental Health Inpatients: A Systematic Review.” Journal of Psychiatric and Mental Health Nursing 29: 220–239. 33835622 10.1111/jpm.12757

[inm70323-bib-0008] Belayneh, Z. , J. Chavulak , D. A. Lee , M. Petrakis , and T. P. Haines . 2024. “Prevalence and Variability of Restrictive Care Practice Use (Physical Restraint, Seclusion and Chemical Restraint) in Adult Mental Health Inpatient Settings: A Systematic Review and Meta‐Analysis.” Journal of Clinical Nursing 33: 1256–1281. 38304928 10.1111/jocn.17041

[inm70323-bib-0009] Birkeland, S. , T. Steinert , R. Whittington , and F. A. Gildberg . 2024. “Abolition of Coercion in Mental Health Services‐A European Survey of Feasibility.” International Journal of Law and Psychiatry 94: 101992. 38763063 10.1016/j.ijlp.2024.101992

[inm70323-bib-0010] Blumer, H. 1986. Symbolic Interactionism. Perspective and Method. University of California Press.

[inm70323-bib-0011] Brooks, E. , D. Lawrence , R. Bagshaw , et al. 2025. “Race, Restriction, Risk and Reconviction: Findings From an England and Wales Medium Secure Cohort.” Psychiatry, Psychology and Law: 1–18. 10.1080/13218719.2025.2521628.

[inm70323-bib-0012] Buongiorno, L. , F. Mele , G. Petroni , et al. 2025. “Cognitive Biases in Forensic Psychiatry: A Scoping Review.” International Journal of Law and Psychiatry 101: 102083. 40049040 10.1016/j.ijlp.2025.102083

[inm70323-bib-0013] Collazos, F. , Á. Malagón‐Amor , I. Falgas‐Bague , et al. 2021. “Treating Immigrant Patients in Psychiatric Emergency Rooms.” Transcultural Psychiatry 58: 126–139. 32281520 10.1177/1363461520916697 PMC7554163

[inm70323-bib-0014] COUNCIL OF EUROPE . 2017. Means of Restraint in Psychiatric Establishments for Adults. Council of Europe.

[inm70323-bib-0015] Craig, E. , C. Leah , J. Dixon , et al. 2025. “From Mental Health Detention to Health Systems Reform: Co‐Producing Policy and Practice Recommendations With Black Men, Their Communities, and Health and Social Care Professionals.” PLOS Ment Health 2: e0000457. 41662107 10.1371/journal.pmen.0000457 PMC12798615

[inm70323-bib-0016] Della Rocca, B. , M. Luciano , R. Bello , et al. 2024. “Use of Coercive Measures in Refugees and Asylum Seekers: A Systematic Review.” International Review of Psychiatry 36: 762–776. 39630177 10.1080/09540261.2024.2335181

[inm70323-bib-0017] Flanagin, A. , T. Frey , and S. L. Christiansen . 2021. “Updated Guidance on the Reporting of Race and Ethnicity in Medical and Science Journals.” JAMA 326: 621–627. 34402850 10.1001/jama.2021.13304

[inm70323-bib-0018] Fluttert, F. , B. VAN Meijel , C. Webster , H. Nijman , A. Bartels , and M. Grypdonck . 2008. “Risk Management by Early Recognition of Warning Signs in Patients in Forensic Psychiatric Care.” Archives of Psychiatric Nursing 22: 208–216. 18640540 10.1016/j.apnu.2007.06.012

[inm70323-bib-0019] Gildberg, F. A. , P. Fristed , G. Makransky , E. H. Moeller , L. D. Nielsen , and S. K. Bradley . 2015. “As Time Goes by: Reasons and Characteristics of Prolonged Episodes of Mechanical Restraint in Forensic Psychiatry.” Journal of Forensic Nursing 11: 41–50. 25622065 10.1097/JFN.0000000000000055

[inm70323-bib-0020] Gildberg, F. A. , and R. Wilson . 2023a. “Scientific Models for Qualitative Research: A Textual Thematic Analysis Coding System ‐ Part 1.” Nurse Researcher 31: 36–42. 10.7748/nr.2023.e1893 37254707

[inm70323-bib-0021] Gildberg, F. A. , and R. Wilson . 2023b. “Scientific Models for Qualitative Research: A Textual Thematic Analysis Coding System‐Part 2.” Nurse Researcher 31: 36–42. 10.7748/nr.2023.e1893 37254707

[inm70323-bib-0022] Hallett, N. , R. Dickinson , E. Eneje , and G. L. Dickens . 2025. “Adverse Mental Health Inpatient Experiences: Qualitative Systematic Review of International Literature.” International Journal of Nursing Studies 161: 104923. 39383709 10.1016/j.ijnurstu.2024.104923

[inm70323-bib-0023] INQUEST . 2023. I Can't Breathe: Race, Death & British Policing. INQUEST.

[inm70323-bib-0024] Jones, R. M. , L. Vangala , F. Farrokhi , et al. 2025. “Static and Dynamic Variables Associated With Inpatient Aggression: A Two‐Year Retrospective Study.” Canadian Journal of Psychiatry 70, no. 8: 620–628. 40415389 10.1177/07067437251343293 PMC12106376

[inm70323-bib-0025] Kennedy, H. G. 2022. “Models of Care in Forensic Psychiatry.” BJPsych Advances 28: 46–59.

[inm70323-bib-0026] Krippendorff, K. 2004. Content Analysis: An Introduction to Its Methodology. Sage.

[inm70323-bib-0027] Macpherson, W. 1999. The Stephen Lawrence Inquiry: Report of an Inquiry by Sir William Macpherson of Cluny. Stationery Office.

[inm70323-bib-0028] Mangaoil, R. A. , K. Cleverley , and E. Peter . 2020. “Immediate Staff Debriefing Following Seclusion or Restraint Use in Inpatient Mental Health Settings: A Scoping Review.” Clinical Nursing Research 29: 479–495. 30051734 10.1177/1054773818791085

[inm70323-bib-0029] Mcgough, S. , D. Wynaden , S. Gower , R. Duggan , and R. Wilson . 2022. “There Is no Health Without Cultural Safety: Why Cultural Safety Matters.” Contemporary Nurse 58: 33–42. 35014602 10.1080/10376178.2022.2027254

[inm70323-bib-0030] Mckenna, K. , B. Paterson , N. Hallett , and L. L. Berring . 2024. “Post‐Occurrence Review.” In Coercion and Violence in Mental Health Settings: Causes, Consequences, Management, edited by N. Hallett , R. Whittington , D. Richter , and E. Eneje . Springer Nature Switzerland.

[inm70323-bib-0031] Ministry of the Interior and Health . 2021. Act on Authorised Healthcare Professionals' Patient Records (Recording, Storage, Disclosure, Transfer, etc.). Retsinformation.

[inm70323-bib-0032] Ministry of the Interior and Health . 2024. Act on the use of restictive practices in mental health, etc. Retsinformation.

[inm70323-bib-0033] Morse, J. , M. Barrett , M. Mayan , K. Olson , and J. Spiers . 2002. “Verification Strategies for Establishing Reliability and Validity in Qualitative Research.” International Journal of Qualitative Methods 1: 1–19.

[inm70323-bib-0034] Muir‐Cochrane, E. , K. Grimmer , A. Gerace , T. Bastiampillai , and C. Oster . 2020. “Prevalence of the Use of Chemical Restraint in the Management of Challenging Behaviours Associated With Adult Mental Health Conditions: A Meta‐Synthesis.” Journal of Psychiatric and Mental Health Nursing 27: 425–445. 31867795 10.1111/jpm.12585

[inm70323-bib-0035] Muir‐Cochrane, E. , and C. Oster . 2021. “Chemical Restraint: A Qualitative Synthesis Review of Adult Service User and Staff Experiences in Mental Health Settings.” Nursing & Health Sciences 23: 325–336. 33605053 10.1111/nhs.12822

[inm70323-bib-0036] Muir‐Cochrane, E. , C. Oster , A. Gerace , S. Dawson , R. Damarell , and K. Grimmer . 2020. “The Effectiveness of Chemical Restraint in Managing Acute Agitation and Aggression: A Systematic Review of Randomized Controlled Trials.” International Journal of Mental Health Nursing 29: 110–126. 31498960 10.1111/inm.12654

[inm70323-bib-0037] Müller, M. , N. Brackmann , M. Jäger , et al. 2023. “Predicting Coercion During the Course of Psychiatric Hospitalizations.” European Psychiatry 66: e22. 36700423 10.1192/j.eurpsy.2023.3 PMC9981454

[inm70323-bib-0038] Mundt, A. P. , E. ROZAS Serri , M. Siebenförcher , et al. 2021. “Changes in National Rates of Psychiatric Beds and Incarceration in Central Eastern Europe and Central Asia From 1990‐2019: A Retrospective Database Analysis.” Lancet Regional Health–Europe 7: 100137. 34557842 10.1016/j.lanepe.2021.100137 PMC8454862

[inm70323-bib-0039] NATIONAL INSTITUTE FOR HEALTH AND CARE EXCELLENCE . 2015. Violence and Aggression: Short Term Management in Mental Health, Health and Community Settings. National Institute for Health and Care Excellence. 40997203

[inm70323-bib-0040] O'brien, B. C. , I. B. Harris , T. J. Beckman , D. A. Reed , and D. A. Cook . 2014. “Standards for Reporting Qualitative Research: A Synthesis of Recommendations.” Academic Medicine 89: 1245–1251. 24979285 10.1097/ACM.0000000000000388

[inm70323-bib-0041] Patel, M. X. , F. N. Sethi , T. R. Barnes , et al. 2018. “Joint BAP NAPICU Evidence‐Based Consensus Guidelines for the Clinical Management of Acute Disturbance: De‐Escalation and Rapid Tranquillisation.” Journal of Psychopharmacology 32: 601–640. 29882463 10.1177/0269881118776738

[inm70323-bib-0042] Paton, C. , C. E. Adams , S. Dye , O. Delgado , C. Okocha , and T. R. E. Barnes . 2019. “Physical Health Monitoring After Rapid Tranquillisation: Clinical Practice in UK Mental Health Services.” Therapeutic Advances in Psychopharmacology 9: 2045125319895839. 31897297 10.1177/2045125319895839 PMC6920590

[inm70323-bib-0043] Pedersen, M. L. , A. Bricca , J. Baker , O. Schjerning , T. Munk‐Olsen , and F. A. Gildberg . 2025a. “Ethnic Disparities in Rapid Tranquillisation Use and Explanations in Adult Mental Health Emergency Settings? A Systematic Review.” General Hospital Psychiatry 95: 93–101. 40328101 10.1016/j.genhosppsych.2025.04.014

[inm70323-bib-0044] Pedersen, M. L. , A. Bricca , J. Baker , O. Schjerning , T. Munk‐Olsen , and F. A. Gildberg . 2025b. “Ethnic Disparities in Rapid Tranquillisation Use and Justifications in Adult Mental Health Inpatient Settings: A Systematic Review and Meta‐Analysis.” BMJ Mental Health 28: e301399. 10.1136/bmjment-2024-301399 PMC11748781 39805639

[inm70323-bib-0045] Pedersen, M. L. , F. Gildberg , J. Baker , J. B. Damsgaard , and E. B. Tingleff . 2022. “Ethnic Disparities in the Use of Restrictive Practices in Adult Mental Health Inpatient Settings: A Scoping Review.” Social Psychiatry and Psychiatric Epidemiology 58: 1–18. 36454269 10.1007/s00127-022-02387-8 PMC9713127

[inm70323-bib-0046] Pedersen, M. L. , F. A. Gildberg , and J. Baker . 2024. “Culturally Appropriate Care and Reduction of Restrictive Practices in Mental Health.” International Journal of Mental Health Nursing 33: 735–736. 38356177 10.1111/inm.13305

[inm70323-bib-0047] Pedersen, M. L. , F. A. Gildberg , and S. Birkeland . 2022. “The Danish Court Case Database: A Data Source in Forensic Mental Health?” Scandinavian Journal of Forensic Science 28: 1–5.

[inm70323-bib-0048] Pedersen, M. L. , F. A. Gildberg , R. Laulund , K. Jørgensen , and E. B. Tingleff . 2023. “Nurses' Clinical Decision‐Making in the Use of Rapid Tranquillization in Adult Mental Health Inpatient Settings: An Integrative Review.” International Journal of Mental Health Nursing 32: 1274–1288. 37341210 10.1111/inm.13181

[inm70323-bib-0049] Riahi, S. , G. Thomson , and J. Duxbury . 2020. “A Hermeneutic Phenomenological Exploration of “last Resort” in the Use of Restraint.” International Journal of Mental Health Nursing 29: 1218–1229. 32691506 10.1111/inm.12761

[inm70323-bib-0050] Schoer, N. , C. W. Huang , and K. K. Anderson . 2019. “Differences in Duration of Untreated Psychosis for Racial and Ethnic Minority Groups With First‐Episode Psychosis: An Updated Systematic Review and Meta‐Analysis.” Social Psychiatry and Psychiatric Epidemiology 54: 1295–1298. 31183503 10.1007/s00127-019-01737-3

[inm70323-bib-0051] Smith, C. M. , N. A. Turner , N. M. Thielman , D. S. Tweedy , J. Egger , and J. P. Gagliardi . 2022. “Association of Black Race With Physical and Chemical Restraint Use Among Patients Undergoing Emergency Psychiatric Evaluation.” Psychiatric Services 73: 730–736. 34932385 10.1176/appi.ps.202100474

[inm70323-bib-0052] Smith, S. , and R. Mohan . 2024. “Tackling Ethnic Inequality in Forensic Mental Healthcare.” In Seminars in Forensic Psychiatry, edited by M. Davoren and H. G. Kennedy . Cambridge University Press.

[inm70323-bib-0053] Steinert, T. , and P. Lepping . 2009. “Legal Provisions and Practice in the Management of Violent Patients. A Case Vignette Study in 16 European Countries.” European Psychiatry 24: 135–141. 18455912 10.1016/j.eurpsy.2008.03.002

[inm70323-bib-0054] Tomlin, J. , I. Lega , P. Braun , et al. 2021. “Forensic Mental Health in Europe: Some Key Figures.” Social Psychiatry and Psychiatric Epidemiology 56: 109–117. 32651594 10.1007/s00127-020-01909-6 PMC7847441

[inm70323-bib-0055] Tully, J. , J. Hafferty , D. Whiting , K. Dean , and S. Fazel . 2024. “Forensic Mental Health: Envisioning a More Empirical Future.” Lancet Psychiatry 11: 934–942. 38945145 10.1016/S2215-0366(24)00164-0

[inm70323-bib-0056] Völlm, B. , and N. Nedopil . 2016. The Use of Coercive Measures in Forensic Psychiatric Care: Legal, Ethical and Practical Challenges. Springer.

[inm70323-bib-0057] Williams, D. R. , and O. S. Etkins . 2021. “Racism and Mental Health.” World Psychiatry 20: 194–195. 34002496 10.1002/wps.20845 PMC8129841

